# Florence Nightingale

**Published:** 1860-03

**Authors:** 


FLORENCE NIGHTINGALE.—Florence Nightingale needs but few prefatory remarks. Reared in luxury, and fitted both by natural and acquired endow-

---

ments to adorn the most elevated social position, her immolation of self and voluntary resignation of those domestic ties which ordinarily constitute the charm of a woman's life, more especially commend her to the world's reverence and praise. Long before the disastrous Crimean war was projected, her gentle ministerings were known throughout Great Britain, Germany, and Egypt, where she attended the sick Arabs. She daily frequented the hospitals and reformatory institutions of London, Edinburgh, and the continent, and, in 1851, went to the great Lutheran hospital at Kaiserwerth, on the Rhine, where she spent months in constant attendance on the patients, and perfected herself in the art of nursing. On her return to England, she undertook the management of the Sanatorium for Governesses, and by her efficient aid, both monetary and personal, saved the institution from the neglect and ruin into which it was fast sinking. Immediately afterwards came accounts of the sufferings in the East, and of the additional hardships which the wounded soldiers were enduring from want of proper care and attention. The English, with their characteristic promptness, soon raised the sum of £15,000, which was expended in articles of food and clothing for the relief of their army. It was soon felt, however, that the sufferings were chiefly to be ascribed to the absence of proper nursing, and, at the instigation of the Hon. Sydney Herbert, Miss Nightingale was selected as the person best calculated to undertake the control of the British establishment for wounded soldiers and sailors in the Crimea. Many would have shrunk from such a proposal, knowing the terrible dangers and toils to which it would necessarily subject them; but Florence Nightingale gloried in the prospect; it was a more extended field for her labors, and one in which her Christian virtues of philanthropy, benevolence, and charity could be exercised to the utmost.

In October, 1854, accompanied by the Rev. Mr. Bricebridge and his wife, and thirty-seven nurses, she left England on her holy and ennobling mission, reaching Scutari in November, just before the wounded of Balaklava were brought into the hospitals. There it was that she proved herself to be eminently capacitated for the arduous duty she had undertaken. Throughout the wards, where confusion had reigned before her arrival, the most perfect order and regulation were maintained; and it was not only while actively engaged for the relief of the sick, that her presence was acknowledged by all to be a blessing. There was many a word of encouragement and smile of sympathy which revived the despondent hearts of the poor sufferers more than any personal aid could possibly have done. In proof of this there is a passage from one of the soldier's letters which, though written in a homely style, is replete with poetry which must appeal to every human heart. In speaking of the pleasure it was to see Miss Nightingale pass, he says: "She would speak to one and to another, and nod and smile to a many more, but she couldn't do it to all, you know; we lay there by hundreds; but we could kiss her shadow as it fell, and lay our heads on the pillows again, content."

After her return from the Crimea, $600,000 were placed at her disposal as a testimony of a nation's gratitude; this sum she appropriated to the erection and management of a Training-School for Nurses, thus endeavoring to perpetuate her own example in others.

Very little has been heard of her for two years, but during this time she has not been idle; in her retirement she has written a hand-book, called "Notes on Nursing; what it is, and what it is not,"—which was issued a month ago in London. This volume is the result of her own experience in her philanthropic career, and by its publication she bestows as great services as she rendered in the hospitals at Scutari.

